# MiR-21 in the Cancers of the Digestive System and Its Potential Role as a Diagnostic, Predictive, and Therapeutic Biomarker

**DOI:** 10.3390/biology10050417

**Published:** 2021-05-08

**Authors:** Ha Thi Nguyen, Salah Eddine Oussama Kacimi, Truc Ly Nguyen, Kamrul Hassan Suman, Roselyn Lemus-Martin, Humaira Saleem, Duy Ngoc Do

**Affiliations:** 1 Institute of Research and Development, Duy Tan University, Danang 550000, Vietnam; nguyenthiha23@duytan.edu.vn; 2 Faculty of Medicine, Duy Tan University, Danang 550000, Vietnam; 3 Faculty of Medicine, University of Tlemcen, Tlemcen 13000, Algeria; kacimi.oussama97@yahoo.com; 4 Department of Agricultural Biotechnology and Research Institute of Agriculture and Life Sciences, Seoul National University, Seoul 08826, Korea; truclyst@snu.ac.kr; 5 Department of Fisheries Biology & Aquatic Environment, Bangabandhu Sheikh Mujibur Rahman Agricultural University, Gazipur 1706, Bangladesh; kamrulhasan2380@gmail.com; 6 HPQC Labs, Waterloo, ON N2T2K9, Canada; roselyn@hpqc.org; 7 Jamil–ur–Rahman Center for Genome Research, Dr. Panjwani Center for Molecular Medicine and Drug Research, International Center for Chemical and Biological Sciences, University of Karachi, Karachi 75270, Pakistan; humaira.saleemsohail@hotmail.com; 8 Department of Animal Science and Aquaculture, Dalhousie University, Truro, NS B2N5E3, Canada

**Keywords:** miR-21, cancer, pancreas, liver, gastrointestinal tract, digestive system

## Abstract

**Simple Summary:**

MicroRNAs are small, non-coding RNA molecules that can regulate the expression of various cancer-related genes and thereby contribute to tumorigenesis and progression of many cancer types. The biological functions and therapeutic potential of miR-21 have been comprehensively investigated. In the current study, we provide an inclusive review of the potential targets, and the current applications as a diagnostic and prognostic cancer biomarker of miR-21. We also summarize the scientific evidence that has highlighted miR-21 as a therapeutic agent as well as the challenges for its use as a therapeutic tool in different cancer types in the digestive system.

**Abstract:**

MicroRNAs (miRNAs) are small non-coding RNAs. They can regulate the expression of their target genes, and thus, their dysregulation significantly contributes to the development of cancer. Growing evidence suggests that miRNAs could be used as cancer biomarkers. As an oncogenic miRNA, the roles of miR-21 as a diagnostic and prognostic biomarker, and its therapeutic applications have been extensively studied. In this review, the roles of miR-21 are first demonstrated via its different molecular networks. Then, a comprehensive review on the potential targets and the current applications as a diagnostic and prognostic cancer biomarker and the therapeutic roles of miR-21 in six different cancers in the digestive system is provided. Lastly, a brief discussion on the challenges for the use of miR-21 as a therapeutic tool for these cancers is added.

## 1. Introduction

MicroRNAs (miRNAs) are a group of small, non-coding RNAs (ncRNAs) of 18–24 nucleotides in length [1]. They can bind to the 3′-untranslated region (3′-UTR) of a target mRNA molecule to exert degradation and/or translation inhibition, thereby regulating the expression of the target genes [2]. MiRNAs, therefore, play essential roles in many physiological and cellular processes, including differentiation, proliferation, and apoptosis [3]. They may function either as oncogenic miRNAs (oncomiRs [4]) or tumor suppression agents [5,6]. Among them, microRNA-21 (miR-21), one of the first identified mammalian miRNAs, has been recognized as an oncomiR that is overexpressed in various human cancer types, including breast, gastric, lung, esophageal, colorectal, biliary tract, nasopharyngeal, and liver cancers, as well as osteosarcoma, glioma, leukemia, retinoblastoma, and lymphoma [7,8]. The conditional over-expression of miR-21 in miR-21 “knock-in” mouse caused malignant B-cell lymphoma has strengthened its function as an oncomiR [9].

MiR-21 is encoded by a genomic sequence that is transcribed by RNA polymerase II to form a primary miRNA (pri-miRNA) of ~3433 nucleotides (nt). Pri-miR-21 is then processed by Drosha and DiGeorge Syndrome critical region 8 (DGCR8) to form a stem-loop precursor (pre-miR-21) of ~72 nt [10]. The pre-miR-21 is then transported into cytoplasm by Epotin-5 and Ran-GTP where it is further trimmed by Dicer to generate two mature forms of 22 nt [11,12,13], namely, miR-21-5p (UAGCUUAUCAGACUGAUGUUGA) and miR-21-3p (CAACACCAGUCGAUGGGCUGU). In human genome, miR-21 encoded gene is localized on the host gene VMP1 (https://rnacentral.org/rna/URS000009262D/9606, accessed on 10 July 2020) on chromosome 17 (chr17:59,838,822-59,842,255 (GRCh38/hg38)) of the human genome [14].

**Figure 1:**
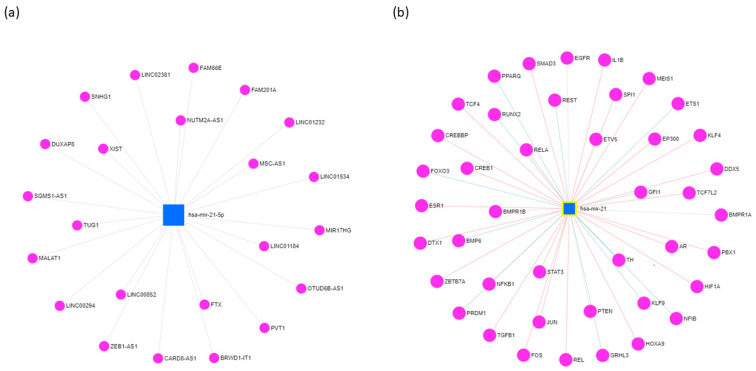
The long non-coding RNAs (**a**) and transcription factors (**b**) interact with miR-21 in various cancer types. The visualization is based on miR-21 targets of the miRNet platform (https://www.mirnet.ca/). Each dot represents a long non-coding RNA or a transcription factor.

Like other miRNAs, miR-21 directly binds to the 3′-UTR of its target genes, and negatively alters their expression. To date, a total of 3078 human genes have been identified as potential targets of miR-21 (https://www.mirnet.ca/; accessed on 20 July 2020), and many of them are involved in regulating different aspects of cancer, such as proliferation, survival, migration, and invasion. Additionally, miR-21 also plays essential roles in the ncRNA interaction network. Specifically, miR-21 modulates the expression of 23 lncRNAs (Figure 1a), including suppressing the expression of growth arrest-specific 5 (GAS5) in the breast tumor specimens, promoting the expression of small nucleolar RNA host gene 1, and activating Akt (protein kinase B) pathway in hepatocellular carcinoma (HCC) cells [15]. Moreover, miR-21 is found to be regulated by 696 circular RNAs (circRNAs) (according to the miRNet database; https://www.mirnet.ca/; accessed on 20 July 2020). For instance, circRNA C3P1 (circC3P1) regulates the miR-21/phosphatase and tensin homolog (PTEN) axis to restrain kidney cancer cell activity [16]; circRNA HIAT1 (circHIAT1) regulates miR-21 to inhibit epithelial–mesenchymal transition (EMT) of gastric cancer (GC) cell lines [17]. Furthermore, epigenetic studies revealed that miR-21 could interact with 42 transcription factors (TFs) (Figure 1b). Ferraro and colleagues, for example, demonstrated that Activator protein 1 (AP-1) and ETS Proto-Oncogene 1 (ETS1) TFs negatively regulate the expression of miR-21 gene by occupying its promoter in a sequence-specific manner [18].

**Figure 2:**
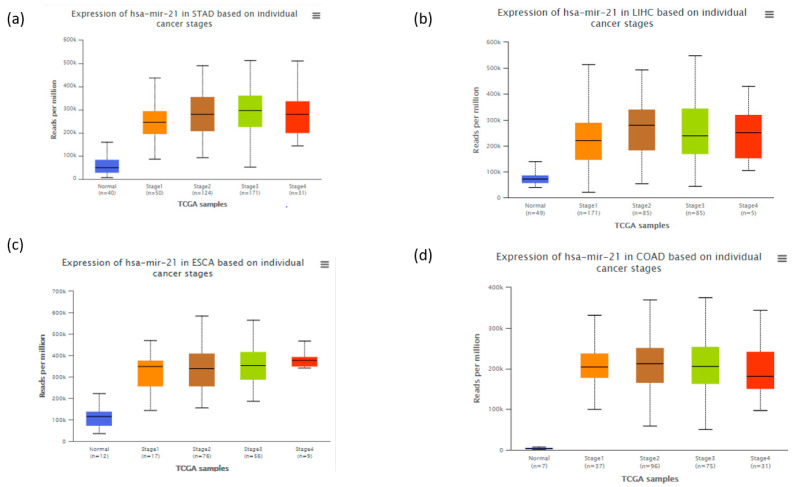
The expression of miR-21 at different stages in four types of cancers (**a**) stomach adenocarcinoma, (**b**) liver hepatocellular carcinoma, (**c**) esophageal carcinoma and (**d**) colon adenocarcinoma. The figure was created based on data obtained from the Cancer Genome Atlas Database and analyzed using UALCAN (http://ualcan.path.uab.edu/analysis-mir.html).

To date, several investigations have focused on the biological functions of miR-21 across human cancers [19,20,21,22,23,24,25,26,27,28,29,30,31]. However, few reviews have been published in the interim, failing to provide a broad view of the topic. In fact, the results obtained from the Cancer Genome Atlas Data (http://ualcan.path.uab.edu/analysis-mir.html, accessed date on 20 April 2020) indicated that miR-21 significantly increased in many cancers types compared to normal tissues including several cancer types of digestive system (Figure 2). Digestive/gastrointestinal cancer refers to malignant tumors that affect both the gastrointestinal tract (GIT) and its accessory glands and are among the most encountered tumors in clinical practice with a high mortality rate [32]. These tumors behave aggressively by locally invading nearby tissues and metastasizing distantly. Among them, colorectal cancer (CRC) is the fourth leading cause of mortality (9.2% of all cases) followed by stomach (8.2%) and liver cancers (8.2%) [33]. In comparison, pancreatic cancer (PC) has the lowest survival rate of all solid tumors with 5-year survival rate of less than 5% [34]. The prognosis for most of the GIT neoplasms is good when the tumor is detected at an early stage. However, these tumors are often diagnosed late due to the expansive nature of the abdomen and lack of less-invasive screening tests or negligence of the patients. Therefore, finding new biomarkers for early diagnosis or screening is of paramount importance for these tumors. In fact, miR-21 significantly increases from stage 1 of cancers (Figure 2), which makes it an interesting candidate for early diagnosis and treatments of cancers.

In this current review, we aimed to provide a comprehensive overview of the roles of miR-21 across cancers of the digestive system, with specific details around its potential use as a biomarker and therapeutic agent in cancer treatments. Additionally, the underlying mechanisms of action of miR-21 in cancers are also discussed.

## 2. Biological Pathways of miR-21 in Cancers of the Digestive System

**Figure 3:**
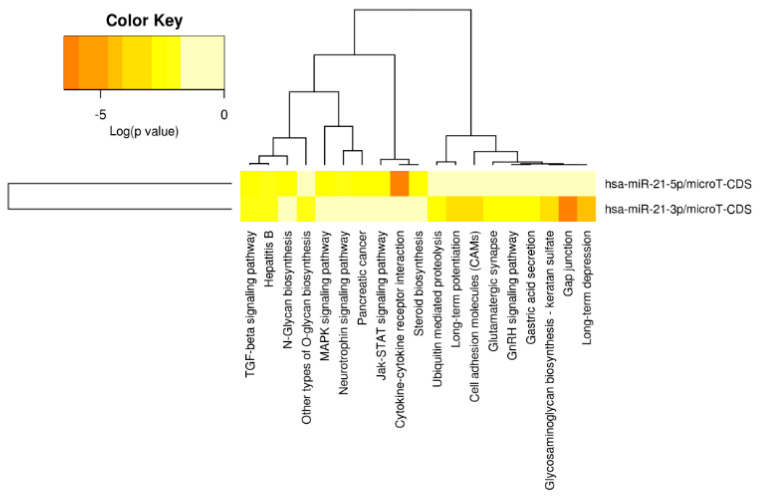
The Kyoto Encyclopedia of Genes and Genomes (KEGG) pathways enriched by miR-21-targeted genes according to the miRPath v.2.0. The color key indicates the log(*p*-value) of the enrichment analyses.

Being a multiple-gene targeted miRNA, miR-21 is expected to be involved in different cellular biological pathways. According to miRPath v.2.0 [35], there are 19 different Kyoto Encyclopedia of Genes and Genomes (KEGG) pathways that were significantly enriched for the target genes of miR-21 (Figure 3). Among those, gap junction, cytokine-receptor interaction, and the transforming growth factor beta (TGF-β) signaling pathway were the three most significantly enriched pathways for miR-21, which have been noted to play roles in cancer progression. Below, we review the major cancer-related pathways induced by miR-21.

### 2.1. Cell Survival and Proliferation

MiR-21 was one of the first reported miRNAs in mammals. Its universal over-expression patterns and its function in human cancers have been well-elucidated. It has been widely established that miR-21 promotes survival and proliferation of cancer cells by directly inhibiting its targets, including *PTEN*, Programmed Cell Death 4 (*PDCD4*), reversion-inducing-cysteine-rich protein with kazal motifs (*RECK*), and sprouty RTK signaling antagonist 2 *(SPRY2*). Accordingly, over-expression of miR-21 in human cancers results in a decreased level of the tumor-suppressor proteins *PTEN*, *PDCD4*, *SPRY2*, and/or *RECK*, thereby promoting cell proliferation [36,37,38,39,40,41,42,43,44,45,46,47]. This effect is achieved via multiple molecular pathways (Figure 3). Particularly, by targeting and downregulating PTEN, miR-21 stimulates the nuclear factor kappa B (NF-κB) [48] or AKT/extracellular signal-regulated kinase (ERK) pathways [42,43], thus inducing cell proliferation and tumorigenesis. In HCC, increased levels of miR-21 downregulate interleukin (IL)-12, its direct target, thus inhibiting apoptosis and promoting cell proliferation [49]. Moreover, induced expression of miR-21 high-mobility group box 1 (HMGB1) causes downregulation of its targets, TIMP metallopeptidase inhibitor 3 (*TIMP3*) and *RECK* that, in turn, increase the levels of matrix metallopeptidase (MMP) proteins, thereby mediating HCC progression and metastases [50]. This study indicated that the signal transducer and activator of transcription 3 (IL-6/STAT3)–miR-21 axis is a novel mechanism through which HMGB1 promotes HCC progression [50]. In PC, it was also demonstrated that miR-21 enhances endothelial growth factor (EGF)-induced proliferation via targeting of *SPRY2* and activating the MAPK/ERK and phosphatidylinositol 3-kinase (PI3K)/AKT signaling pathways [41], revealing a novel potential therapeutic target for patients with PC. Similarly, downregulation of miR-21 consequently inhibits the proliferation, invasion, and migration of esophageal squamous cell carcinoma (ESCC) cells by negatively regulating the expression of PDCD4 [51] or PTEN [45] via the PTEN/PI3K/AKT signaling pathway [46]. In human salivary adenoid cystic carcinoma (SACC), higher expression of miR-21 is linked with higher metastatic potential (SACC-LM cells). This phenotype can be abolished by transfecting SACC-LM cells with a miR-21 inhibitor, to reverse its effects on the expression of PDCD4, PTEN, and Bcl-2 [52].

MiR-21 contributes to cell survival by upregulating the expression of different anti-apoptotic proteins. The stabilization of MCL-1 (myeloid cell leukemia 1), a pro-survival protein, is promoted by the hyper-activation of the PI3K/AKT pathway as a result of PTEN downregulation in miR-21-overexpressed cancer cells [53]. Moreover, the expression of Survivin, another well-known apoptotic inhibitor that is usually upregulated in malignant tissues, was also found to be negatively correlated with the expression of PTEN [38]. Thus, by inhibiting the expression of PTEN, miR-21 upregulates the expression of the anti-apoptotic proteins MCL-1 and/or Survivin, and consequently promotes cancer cell survival. Additionally, the induced expression of Bcl-2 via direct interaction with miR-21 and its association with anti-apoptosis and chemoresistance to gemcitabine of PC cells has also been reported [54]. However, the underlying mechanism of how Bcl-2 is induced by miR-21 remains unclear [55].

### 2.2. Migration and Invasion

**Figure 4:**
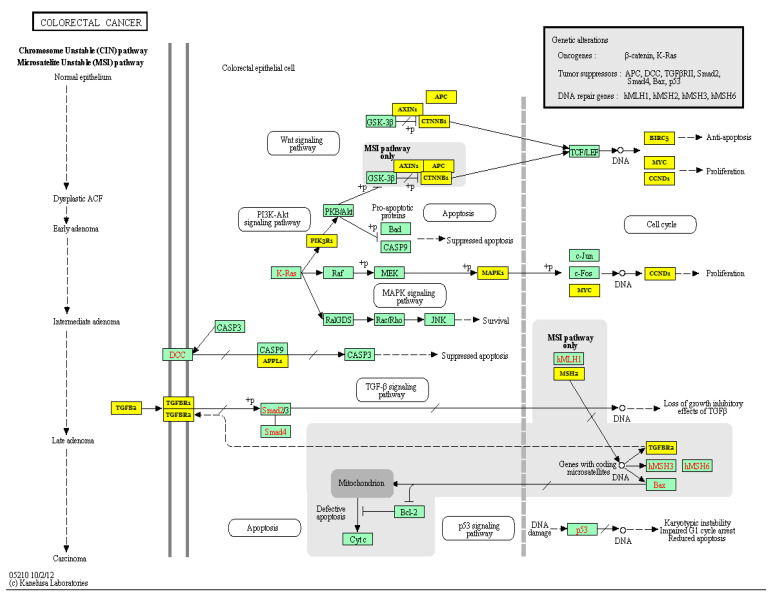
The colorectal cancer pathway and miR-21 target genes. Yellow denotes miR-21-targeted genes according to the miRPath v.2.0.

MiR-21 has also been linked to human tumor invasion and metastasis by negatively regulating targets that are adversely associated with metastatic capacity, such as *PTEN, PDCD4,* von Hippel-Lindau (*VHL*), *TIMP3*, Tropomyosin 1 (*TPM1*), and Serpin Family B Member 1 (*SEPINB1*) [40,47,56,57,58,59,60]. In PC, miR-21 contributes to tumor growth, invasion, and chemoresistance by positively regulating the expression of invasion-related genes including MMP-2/9 and vascular endothelial growth factor (VEGF) [60], and negatively regulating the expression of the tumor-suppressor gene, *VHL* [59]. Inhibition of miR-21 causes upregulation of VHL [59], which inhibits the expression of MMP-2/9 and hypoxia-inducible factor (HIF)-1α/the VEGF pathway, which consequently inhibits the progression and invasion of PC cells [59]. This study demonstrated the oncogenic roles of miR-21 and suggested the miR-21–VHL axis as a potential target for PC therapy. In HCC, on the other hand, downregulation of PTEN due to over-expression of miR-21 causes phosphorylation of FAK (focal adhesion kinase) and over-expression of MMP-2/9, thereby contributing to tumorigenesis [61]. Additionally, downregulation of *PTEN* and human sulfatase-1 (hSulf-1) as a result of increased miR-21 expression triggers the activation of the AKT/ERK pathways and facilitates tumor growth and metastasis in HCC [43]. In CRC, miR-21 promotes tumor invasion and metastasis via modulating the expression of multiple cancer-related genes, including Transforming Growth Factor Beta Receptor 2 (*TGFβR2*) [56], *PDCD4* [57,58], and *PTEN* [40] (Figure 4). Additionally, miR-21 controls the expression of the integrin β4 subunit (*ITGβ4*) that plays a role in regulating the EMT, thereby affecting the migration properties of cancer cells [18]. Moreover, by negatively regulating a tumor suppressor gene, Ras Homolog Family Member B (RhoB), miR-21 promotes cell proliferation, invasion, and apoptosis [62]. Additionally, downregulation of miR-21 in SACC indirectly leads to downregulation of the p-STAT3 protein by upregulating its inhibitor, PDCD4 [44]. STAT3 is involved in multiple fundamental events of cancer pathogenesis, including survival, proliferation, invasion, and angiogenesis, via its target genes, such as Bcl2, c-myc, cyclinD1, Survivin, and MMP-2/9 [63]. Thus, downregulation of STAT3 directly or indirectly via controlling the expression of miR-21 and/or PDCD4 in SACC may eventually inhibit tumor growth and invasion [63]. Furthermore, the reduction of PTEN expression in SACC cells and its negative association with migratory and invasive capacities in vitro and tumor size in vivo have also been reported [64]. Taken together, these results indicate the significance of miR-21 across human gastrointestinal cancers, and its potential as a target for cancer therapy.

### 2.3. Immune Response, Inflammation, and Angiogenesis

MiR-21 is found to be abundantly expressed in multiple cell types including macrophages [65] and T lymphocytes [66]. By inhibiting the expression of its multiple target effectors, miR-21 contributes to the carcinogenesis [67] as well as other cancer-related biological pathways, including immune responses and inflammation. The upregulation of miR-21 upon inflammatory response of macrophages has been reported in both hematopoietic and immune cells [68]. Additionally, a recent study showed the over-expression of miR-21 in macrophages led to a shift towards a pro-inflammatory phenotype, and exosomes’ delivery of miRNAs to naive macrophages also caused an induction of pro-inflammatory markers tumor necrosis factor α (TNFα), IL-1β, inducible nitric oxide synthase (iNOS), and IL-6 and repression of anti-inflammatory cytokine IL-4 [69]. Inhibition of miR-21 expression in tumor-associated macrophages, on the other hand, induced an anti-tumoral immune response by improving cytotoxic T-cell responses via the stimulation of cytokines IL-12 and chemokine 10. This, in turn, promoted tumor cell death and inhibited tumor neovascularization, thereby decreasing tumor growth [60]). The anti-inflammatory function of miR-21 has also been achieved in macrophages in response to bacterial lipopolysaccharide via negative regulation of PDCD4, an inhibitor of IL-10 [70].

**Figure 5:**
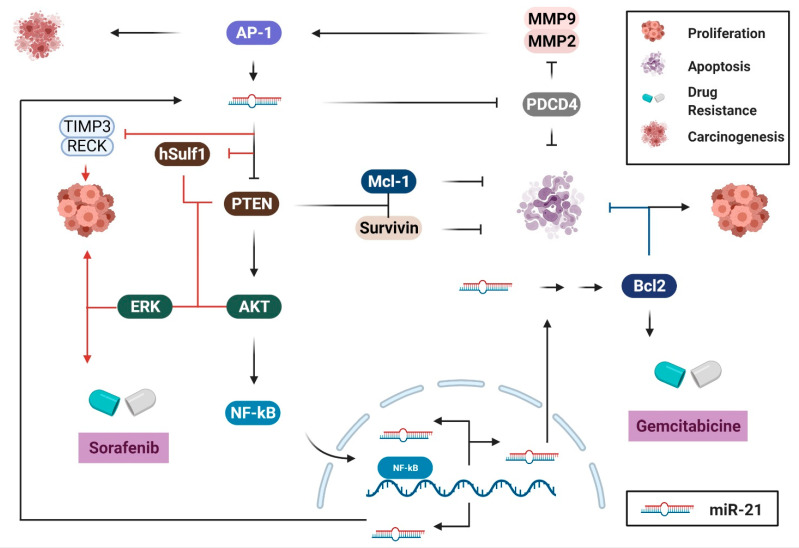
Biological pathways of miR-21 in gastrointestinal cancers. MiR-21 is commonly found to be overexpressed in human cancer, which causes the downregulation of its target genes, including phosphatase and tensin homolog (*PTEN*), Programmed Cell Death 4 (*PDCD4*), reversion-inducing-cysteine-rich protein with kazal motifs (*RECK*), sprouty RTK signaling antagonist 2 (*SPRY2*), and von Hippel-Lindau (*VHL*), thereby promoting survival, proliferation, migration, and metastasis of cancer cells. This figure was made using www.biorender.com.

By mediating the proliferation and migration of vascular cells including smooth muscle cells and endothelial cells, miR-21 is involved in regulating angiogenesis [71,72]. Over-expression of miR-21 in DU145 cells resulted in elevated expression of HIF-1α and VEGF via PTEN/PI3K/AKT and MAPK signaling pathways, which ultimately promoted angiogenesis [73]. A direct interaction between miR-21 and the 3′-UTR of RhoB, a tumor suppressor gene that may be engaged in angiogenesis regulation of HCC lines, was identified [74]. Accordingly, suppression of miR-21 is associated with an elevation of RhoB that leads to the restricted proliferation, migration, and invasion of HCC and metastatic breast cancer cell lines [74]. In contrast, the study of Sabatel and colleagues (2011) in endothelial cells reported miR-21 as a negative modulator of angiogenesis. Specifically, over-expression of miR-21 caused a reduction of RhoB that disturbs endothelial cell migration and tubulogenesis, thereby suppressing angiogenesis [75]. These contradictory results could be due to the nature of two different models that need to be clarified in future studies. Additionally, several studies have demonstrated that reduced expression of FASLG, another direct target of miR-21, could affect cell apoptosis and proliferation in various types of cancers, including breast, CRC, and HCC [76,77] (Figure 5).

## 3. Role of mir-21 as a Diagnostic, Predictive, and Therapeutic Biomarker across Cancers in Digestive System

### 3.1. Gastric Cancer

#### 3.1.1. MiR-21 as a Diagnostic Biomarker in Gastric Cancer

The role of miR-21 as a diagnostic biomarker of GC has been widely investigated, and inconsistent outcomes have been reported [78,79,80]. Different studies have reported a significantly high concentration of miR-21 in the tumor tissue and plasma of GC patients compared to normal individuals [80,81,82,83,84]. Li et al. measured the level of miR-21 in 10 GC patients and 10 healthy control participants using the quantitative real-time polymerase chain reaction (qRT-PCR) method and found a significantly increased plasma level of miR-21 in patients with stage I GC compared to healthy controls [78]. This finding was in accordance with a previous study that suggested high expression levels of miR-21 in GC plasma and primary GC tissue [81]. In that study, the authors analyzed the possibility of detecting miR-21 in plasma samples, then compared plasma miRNA levels between pre- and post-operative paired samples from 10 GC patients. Their results showed the possibility of detection of plasma miR-21 in GC, and its significant reduction post-operatively [81]. Similarly, a significantly increased level of miR-21 was observed in the plasma [80] and peripheral blood mononuclear cells of GC patients compared to healthy individuals [82].

A meta-analysis of five studies with a total of 251 GC patients and 184 controls yielded a moderate pooled sensitivity (66.5%) and specificity (83.1%), suggesting the potential diagnostic value of miR-21 in GC [79]. More recently, a systematic review of the expression profile reported the dysregulation of 97 miRNAs in either the blood or tissue samples of GC patients. Among these, miR-21 and 12 other miRNAs were consistently upregulated in these patients [82]. These findings strongly indicate that miR-21 is an excellent diagnostic biomarker candidate of GC. Further studies are required to validate and strengthen the evidence of its diagnostic value since (i) most of the current evidence comes from studies on Chinese and Japanese populations [79], and (ii) there is limited number of studies and small sample sizes.

#### 3.1.2. MiR-21 as a Prognostic and Predictive Biomarker in Gastric Cancer

Despite advances in cancer management, GC is still one of the most aggressive cancers. Due to the expansive nature of the stomach, most of the time GC is detected at an advanced stage. Different studies have suggested miR-21 as a prognostic factor for patients with GC. Specifically, a higher miR-21 level was reported to be related to a lower overall survival (OS) rate of GC patients [85]. In contrast, the OS rates were significantly improved in GC patients with a lower expression level of miR-21 [86,87]. A meta-analysis study that combined the data of eight independent studies showed a pooled hazard ratio of a higher miR-21 level in tissue samples of 2.00 (95% CI: 1.39–2.88, *p* < 0.01), indicating a significant predictive value of miR-21 for the poorer OS of these patients [88]. Additionally, higher miR-21 levels were associated with worse tumor differentiation and positively associated with lymph node metastasis and tumor-node-metastasis stage [88]. Similarly, Song et al. demonstrated that high levels of miR-21 were associated with an increased tumor size and an advanced T stage, suggesting its role as a biomarker for patients with GC [89].

The role of miR-21 as a potential predictive biomarker in GC has also been investigated. Park et al., for example, reported that miR-21-5p was more highly expressed in the recurrence than in the non-recurrence GC patients among validation samples, with 86.7% sensitivity and 65.5% specificity [90]. Two other studies investigated the role of miR-21 in predicting peritoneal recurrence, which frequently occurs and is associated with poor prognosis, and confirmed a significant association between high miR-21 expression level and peritoneal recurrence in patients with GC [91,92]. The results suggested that (exosomal) miR-21 may serve as a predictive biomarker of peritoneal recurrence in GC.

Besides complete surgical resection, which can efficiently cure patients with early GC, intraperitoneal chemotherapy (IC) is widely used for the treatment of patients with unresectable or recurrent GC. However, there is insufficient evidence on the efficacy of this regimen due to difficulty reporting and predicting the response of the peritoneal recurrence. To investigate the predictive value of miR-21 on tumor response to IC in patients with GC, Ohzawa et al. determined the expression level of exosomal miR-21-5p in peritoneal lavage fluid of 74 patients with advanced GC [93]. The study revealed a significant upregulation of miR-21-5p in patients with peritoneal metastases, which is associated with worse OS than those with lower expression, suggesting its role in modifying chemosensitivity against IC [93]. Kim et al., on the other hand, performed a validation study of circulating miRNA biomarkers, including miR-21, for the prediction of lymph node metastasis in GC, and concluded that miR-21 obtained from different samples could be biomarker candidates to predict recurrence and the presence of lymph nodes and peritoneal metastasis of GC [94].

#### 3.1.3. MiR-21 as a Therapeutic Target in Gastric Cancer

According to the last updated European Society for Medical Oncology (ESMO) clinical practice guidelines for the treatment of cancer, 5-fluorouracil (5-FU), along with other agents, is the first line perioperative chemotherapy regimen for patients with stage I.B resectable GC [95]. Several studies have shown that miRNAs, including miR-21, might be involved in the tumor resistance mechanisms of 5-FU by decreasing the expression levels of their target genes, suggesting anti-miRNA-21 (AMO-21) therapies may play a pivotal role in cancer treatment [96,97]. Indeed, downregulation of miR-21 increases the sensitivity of human epidermal growth factor receptor 2 (HER2)-positive GC in response to both 5-FU and trastuzumab by upregulating target genes of miR-21, *SPRY2*, and PTEN, respectively [98]. Cisplatin is another first-line chemotherapy agent that has been frequently studied. Zheng and colleagues have noted that exosomal miR-21 that is directly transferred from tumor-associated macrophages to the GC cells may confer cisplatin-resistance in GC by suppressing cell apoptosis and activating the PI3K/AKT signaling pathway, which in turn is achieved by downregulation of PTEN expression [96,97]. Additionally, the role of miR-21-5p in doxorubicin (DOX) and trastuzumab (a HER2-targeting monoclonal antibody) resistant GC has also been studied. In both cases, targeting miR-21 was reported to be an effective therapy to reverse resistance in GC cells [99].

### 3.2. Colorectal Cancer

#### 3.2.1. MiR-21 as a Potential Prognostic and Predictive Biomarker in Colorectal Cancer

Scientific evidence has suggested the role of miR-21 as a biomarker in CRC. Toiyama and colleagues showed that serum miR-21 levels robustly distinguished adenoma and CRC, suggesting its potential role in early colon cancer detection [100]. Moreover, it has been indicated that the miR-21-5p level is strongly associated with stage II colon cancer mortality [101]. The authors suggested using miR-21-5p and a high inflammatory risk score (IRS) in combination for predicting unfavorable outcomes of colon cancers, especially for stage II. Similarly, a retrospective study dissecting the association of seven miRNAs with stage II CRC outcomes in a Chinese population also advised using miR-21-5p in combination with other indicators to enhance the prognostic accuracy for stage II CRC [102]. In another study, Nielsen et al. reported that miR-21-5p could predict the outcome of patients with colon cancer, but not for those with rectal cancer [103]. Another group conducted a meta-analysis of the prognostic roles of miR-21 in CRC cancers based on seven selected studies [104] and reported a significant correlation of miR-21 level and stage III/IV patients’ survival, indicating the potential role of miR-21 as a predictive biomarker in CRC. Nevertheless, it is generally agreed that a set of several miRNAs including miR-21 offers better prediction of CRC outcome than miR-21 alone.

#### 3.2.2. Roles of miR-21 in Chemoresistance in Colorectal Cancer

Growing evidence suggests the use of miR-21 alone or in combination with other miRNAs in predicting response to chemoradiotherapy (CRT) in CRC [105,106,107]. A significant association between miR-21 in pre-neoadjuvant CRT tumor tissue and response, with a 3.67 odds ratio (OR) of incomplete response in patients with higher miR-21 levels (*p* = 0.04), has been reported, suggesting the role of miR-21 in predicting an incomplete response to CRT in rectal adenocarcinoma [106]. Over-expression of miR-21 has been noted to be associated with 5-FU resistance by targeting and inhibiting the expression of its direct target gene, *PDCD4* [108], and miR-21 has been proposed to be used as an important indicator for 5-FU therapeutic efficacy in CRC [109]. The study by Liang et al. [110] using a combination of 5-FU and miR-21 inhibitor oligonucleotide (miR-21i) to explore the roles of miR-21 in inducing chemoresistance in CRC cells showed that the combination has not only reversed chemotherapy resistance but also enhanced cytotoxicity of 5-FU on these cells. On the other hand, Chen et al. [111], investigated the possible role of miR-21 in topoisomerase-inhibitors resistance and found that over-expression of miR-21 induced resistance to topoisomerase inhibitors without an alteration in topoisomerase activity.

#### 3.2.3. MiR-21 as a Potential Therapeutic Biomarker in Colorectal Cancer

Despite advances in cancer research and emergence of targeted and immunotherapies, the mortality rate of patients with CRC remains high, especially for those with later stages. Surgical resection of the tumor is a common approach to treat local forms while chemotherapy or other adjuvant therapies are generally applied when the tumor already invades surrounding tissue and/or metastasizes. Nonetheless, the mortality rate of patients at these stages remains high due to the risk of resistance to the therapies. In the network of miRNAs and pathological pathways, targeting miR-21 seems promising in term of reversing chemotherapy resistance. Currently, an interventional clinical trial is being conducted to test the use of an miRNA tool, including six miRNAs (miR-21, miR-20a-5p, miR-103a-3p, miR-106b-5p, miR-143-5p, and miR-215), to determine whether a patient with stage II colon cancer should not receive adjuvant chemotherapy (ClinicalTrials.gov: NCT02466113) according to OS and disease-free survival (DFS) [112].

### 3.3. Pancreatic Cancer

#### 3.3.1. MiR-21 as a Potential Diagnostic Biomarker in Pancreatic Cancer

Szafranska et al. [113] characterized the miRNome in normal and pancreatic ductal carcinoma (PDC) tissues, and found that various miRNAs, including miR-21 were over-expressed in PC cells. This finding revealed miR-21 as a critical potential diagnostic biomarker and therapeutic target in PC. Recent studies have established abnormal miRNA expression in precursor lesions of PC, which reinforces the observations of miRNAs in different stages of the carcinogenic process [114,115]. Additionally, the serum levels of various miRNAs can distinguish cancer patients from healthy individuals, positioning them as potential novel biomarkers for the early detection of PC [116,117].

#### 3.3.2. Roles of miR-21 in Chemoresistance and Regulation of Apoptosis

It has been concretely demonstrated that multiple miRNAs modify cellular responses to anticancer drugs by altering the cell cycle and apoptotic response [118]. Over-expression of anti-apoptotic proteins that allow cancer cells to avoid the apoptotic process is considered to be one of the main causes of chemotherapeutic resistance. By direct upregulation of Bcl-2, miR-21 leads to apoptosis-associated chemoresistance to gemcitabine and consequently, proliferation of PC cells [54]. The PI3K/Akt pathway, on the other hand, plays a role in balancing pro-apoptotic and anti-apoptotic signals, which determines a cell’s survival. Increased miR-21 expression has been reported to be associated with the activation of this pathway. A combination of anti-miR-21 treatment with drugs that target the PI3K/AKT/mechanistic target of the rapamycin (mTOR) pathway, such as gemcitabine, reduces the level of pAKT and intensifies apoptosis by increasing the apoptosis induction of this chemotherapy drug [119,120] (Figure 5). The anti-apoptotic role of miR-21 might be specific to certain cancers, such as pancreatic and bile duct cancers, which reinforces the focus on miR-21 as a target for PC. Meng et al. alternatively reported that gemcitabine-induced apoptosis is inhibited by miR-21 via targeting PTEN [121]. Nevertheless, Moriyama et al. [60] did not observe differences in the PTEN expression levels in PC compared to normal cells. These findings indicate the need for further studies to identify the specific target genes of miR-21, and the molecular pathways associated with chemoresistance in PC.

### 3.4. Liver Cancer

#### 3.4.1. MiR-21 as a Prognostic and Diagnostic Biomarker in Liver Cancer

There is a strong association between miR-21 expression and the prognosis of HCC [122]. Particularly, miR-21 is an established survival factor in HCC, and increased expression of miR-21 is significantly associated with tumor progression. However, the use of miR-21 as a diagnostic and therapeutic target in liver cancer is controversial and requires further study [123]. Wang et al. observed a poor prognosis of HCC patients with high expression of miR-21, proposing the potential use of miR-21 as a prognostic biomarker in patients with HCC [124].

#### 3.4.2. MiR-21 as a Therapeutic Target in Liver Cancer

Downregulation of *RECK*, *PTEN*, and *PDCD4* due to the over-expression of miR-21 in HCC results in high levels of MMPs, which facilitate tumor progression and metastases. Therefore, pharmaceutical approaches targeting miR-21 alone or in combination with other chemotherapy agents could possibly be a therapeutic option for HCC [125]. The Kruppel-like factor 5 (*KLF5*) gene has been reported to function as a tumor suppressor, which inhibits cell invasion and migration in numerous types of cancers [126]. MiR-21 inhibits *KLF5* gene expression by binding to its 3′-UTR, thereby promoting HCC cell migration and invasion. The miR-21/KLF5 axis approach, therefore, could also be a useful therapeutic target for HCC treatment [127].

In HCC, miR-21 contributes to sorafenib resistance via the PTEN/Akt pathway. By overcoming sorafenib resistance, miR-21 could serve as a therapeutic target in the treatment of HCC [128]. In fact, anti-miR-21, a specific and potent single-stranded oligonucleotide, and miR-21 inhibitors, are being used as promising therapeutic targets for the treatment of liver cancer [129]. HCC cells transfected with anti-miR-21 combined with 5-FU and interferon-*α* administration have been shown to significantly increase sensitivity to chemotherapy [130]. Wagenaar et al. conducted an in vivo study using a broad panel of HCC cell lines and evaluated the effect of specific single-strand oligonucleotide inhibitors of miR-21 on Dimethylarginine Dimethylaminohydrolase 1 (*DDAH1*), Ankyrin Repeat Domain 46 (*ANKRD46*), and *RECK* gene expression, and found that inhibition of miR-21 hinders the growth and proliferation of HCC cells. The results indicate that miR-21 can be used as a potential target for HCC therapy [129].

### 3.5. Salivary Gland Cancer

#### 3.5.1. MiR-21 as a Prognostic and Diagnostic Biomarker in Salivary Gland Cancer

Due to the presence of multiple potential cancer biomarkers, analysis of saliva has been demonstrated as an effective diagnostic approach for various distant cancers [131]. Salivary miR-21 has also emerged as a promising biomarker for the detection of different types of cancer [132]. An increased level of miR-21 has been reported in human SACC in several studies [44,52], indicating its role in the growth and metastasis of these cells and its potential to be used as a diagnostic biomarker for this type of cancer [44,52]. However, the role of miR-21 as a prognostic and diagnostic biomarker for salivary gland cancer remains to be clarified.

#### 3.5.2. MiR-21 as a Therapeutic Target in Salivary Gland Cancer

The treatment of salivary gland cancer (SGC) patients involves radiotherapy and/or an operation, due to the limitations of drugs [133]. Frequent changes of the *HER2* gene and its positive response toward HER2-driven therapy have revealed bridges between salivary duct carcinomas (SDC) and breast intraductal carcinomas. Such a resemblance indicates the potential use of HER2-related breast cancer treatments for SDC patients [134]). Additionally, positive outcomes in treating SGC patients with trastuzumab emtansine (T-DM1), neratinib, vemurafenib, entrectinib, and larotrectinib have also been noted [135,136]. The function of miR-21 in the SACC was indicated by effective inhibition of SACC progression by combination of simvastatin and miR-21 inhibitors [137].

### 3.6. Esophageal Cancer

#### 3.6.1. MiR-21 as a Diagnostic Biomarker in Esophageal Cancer

Due to its atypical symptomatology, the majority of patients with esophageal cancer (EC) are diagnosed at advanced stages, leading to worse outcomes and high mortality [138]. Therefore, the discovery of tumor biomarkers to aid with early detection and better prognosis for this cancer is urgently needed. Several miRNAs, including miR-21, appear to be a great tool for this application. In this context, a meta-analysis of 33 miRNAs, including miR-21, yielded a pooled sensitivity and specificity of 0.79 (95% confidence interval, 0.76–0.82 for both). Accordingly, plasma miR-21, miR-223, and miR-375 may be potential non-invasive diagnostic biomarkers in patients with early-stage ESCC [139]. Similarly, Ye et al. compared the expression and early diagnostic value of salivary and plasma miR-21 in EC and revealed that both salivary and plasma miR-21 are over-expressed in EC tissues compared to control groups, and both can be sensitive biomarkers in EC [140].

#### 3.6.2. MiR-21 as a Prognostic and Predictive Biomarker in Esophageal Cancer

EC is a type of cancer characterized by its high mortality rate and poor prognosis at the time of diagnosis. In this context, several studies have highlighted the potential role of miR-21 as a prognostic biomarker in digestive cancers, including EC. These studies were recently summarized in a systematic review and meta-analysis, highlighting the prognostic significance of circulating miR-21 in esophageal, pancreatic, and CRC. The meta-analysis of two studies reporting patients with ESCC showed that upregulation of miR-21 was linked to worse OS, with a pooled hazard ratio HR of 3.49 (95% CI 2.58–4.71, *p*-value < 0.01). Although the meta-analysis is limited by the small number of studies included, the present evidence shows that miR-21 could be one of the best prognostic biomarker candidates [141].

On the other hand, only a few studies have investigated the role of miR-21 as a predictive biomarker for chemoresistance in ESCC. For this purpose, Komatsu et al. tested whether circulating miR-21 can predict and promote chemoresistance in patients with ESCC [142]. The study showed high pre-treatment plasma concentrations of miR-21 in ESCC patients treated with cisplatin and 5-FU, with a common histopathological response compared to those with a high histopathological treatment response (*p* = 0.0416) [142]. Further studies are required to elaborate the potential role of miR-21 as a predictive biomarker in EC.

#### 3.6.3. MiR-21 as a Therapeutic Target in Esophageal Cancer

The treatment of EC requires multidisciplinary team management, and the optimal treatment option is still controversial. To date, multimodal treatment, including chemotherapy and radiation therapy with or without surgery, is the main treatment option for EC patients [143]. However, treatment resistance remains a big concern in EC. Hence, determining resistance-associated factors in patients with EC is crucial. Incidentally, miR-21 has been found to play a major role in both chemotherapy and radiotherapy resistance of ESCC. Yang et al. reported that the over-expression of miR-21 significantly decreased the sensitivity of ESCC cells to cisplatin by negatively controlling the expression of PDCD4 [144]. Over-expression of miR-21 was noted to be positively correlated with advanced clinical stage [145] and increased radiation-resistance by increasing cell proliferation and invasion and inhibiting apoptosis of ESCC cells [146]. Together, these studies suggest that miR-21 might be a promising novel target for developing personalized treatments for EC patients in the near future.

## 4. Perspectives of miR-21 in Digestive Tract Cancers

The potential use of miRNAs as biomarkers in cancers has been intensively reviewed [147,148,149]. Thanks to their high specificity to tissues and cell types, some miRNAs have been successfully used to discriminate disease stages and monitor responsiveness to therapies [150,151]. Since miRNAs are more stable than other nucleic acids under a wide range of conditions, they can be extracted from a variety of liquid biospecimens and tissue samples, making them ideal biomarker candidates. Although miR-21 has been proposed as a plausible diagnostic and predictive biomarker and a therapeutic target for several types of cancer, some limitations still exist, for example, its dysregulation has been linked to more than one type of cancer [152]; and a similar expression level of miR-21 has been noted between benign injury and malign tumor. As a result, although miR-21 levels in plasma significantly increased in CRC patients, this candidate biomarker could not be used to distinguish the carcinoma and benign polyps [153]. This finding strongly suggests that a strict process of screening is required before the translation of any miRNAs from bench to bedside.

The pathology behind cancers of the digestive tract is known to be associated with the microorganisms that colonize it [154,155]. The significant correlation between the specific groups of bacteria or taxa in the tumor microenvironment and the number of differentially expressed genes in CRC suggests that miRNAs may stimulate host–microbe interactions. The host–microbiome interaction mechanisms are important to explain the connection of dysbiosis with chronic inflammation and processes that influence carcinogenesis and tumor progression in colon cancer [156]. In the stomach, Helicobacter pylori is a well-known contributor to carcinogenesis [157]. Additionally, miRNA–microbiota interaction is essential for gut homeostasis and CRC [158]. MiR-21 has been reported to play essential roles in some microbiota in GIT-related diseases and cancers [159,160,161]. Commensal bacteria increase the miR-21-5p expression level and promote the permeability of intestinal epithelial cells [162].

The gut–brain axis has recently emerged as a new paradigm in both oncology and neuroscience, particularly for its crucial role in tumorigenesis and the development of cancers [163]. The gut can communicate with the brain hormonally, with gut peptides released from enteroendocrine cells, modulating appetite, while the microbiota modulates neurodevelopment by recruiting different miRNAs. The gut–brain axis represents a communication system that may lead to cancer formation when disrupted. Due to their roles in many biological processes, it is not surprising that miRNAs, including miR-21, may impact the gut–brain axis functions [164]. Further studies assessing the interaction between miR-21 and the microbiota in general, and the gut–brain axis in particular, are required to develop more efficient diagnostic tools and treatment methods against cancers in the digestive tract.

## 5. Conclusions

It has been well-established that miR-21 plays a role in the pathogenesis of digestive tract cancers. Accumulated evidence shows that miR-21 mostly acts as a tumor suppressor that inhibits cell proliferation, invasion, metastasis, and tumor growth in different types of cancer. Various target genes and pathways have been associated with miR-21 functions. The possible interactions of miRNAs with other non-coding RNAs are being established. Further investigations into the sensitivity and specificity of miR-21 as a diagnostic biomarker, adverse off-target effects of using anti-miR-21 as a therapeutic approach, and so forth are required. A deeper understanding of miR-21, its target genes, and the molecular mechanisms of action will allow a successful translation of the current research into clinical applications.

## Data Availability

No data required in this review.
